# Dual processing model of medical decision-making

**DOI:** 10.1186/1472-6947-12-94

**Published:** 2012-09-03

**Authors:** Benjamin Djulbegovic, Iztok Hozo, Jason Beckstead, Athanasios Tsalatsanis, Stephen G Pauker

**Affiliations:** 1 Center for Evidence-based Medicine and Health Outcomes Research, Tampa, FL, USA; 2 Department of Internal Medicine, Division of Evidence-based Medicine and Health Outcomes Research University of South Florida, Tampa, FL, USA; 3 Departments of Hematology and Health Outcomes and Behavior, H. Lee Moffitt Cancer Center & Research Institute, Tampa, FL, USA; 4 Department of Mathematics, Indiana University Northwest, Gary, IN, USA; 5 College of Nursing, University of South Florida, Tampa, FL, USA; 6 Division of Clinical Decision Making, Department of Medicine, Tufts Medical Center, Boston, USA; 7 USF Health, 12901 Bruce B. Downs Boulevard, MDC27, Tampa, FL, 33612, USA

## Abstract

**Background:**

Dual processing theory of human cognition postulates that reasoning and decision-making can be described as a function of both an intuitive, experiential, affective system (system I) and/or an analytical, deliberative (system II) processing system. To date no formal descriptive model of medical decision-making based on dual processing theory has been developed. Here we postulate such a model and apply it to a common clinical situation: whether treatment should be administered to the patient who may or may not have a disease.

**Methods:**

We developed a mathematical model in which we linked a recently proposed descriptive psychological model of cognition with the threshold model of medical decision-making and show how this approach can be used to better understand decision-making at the bedside and explain the widespread variation in treatments observed in clinical practice.

**Results:**

We show that physician’s beliefs about whether to treat at higher (lower) probability levels compared to the prescriptive therapeutic thresholds obtained via system II processing is moderated by system I and the ratio of benefit and harms as evaluated by both system I and II. Under some conditions, the system I decision maker’s threshold may dramatically drop below the expected utility threshold derived by system II. This can explain the overtreatment often seen in the contemporary practice. The opposite can also occur as in the situations where empirical evidence is considered unreliable, or when cognitive processes of decision-makers are biased through recent experience: the threshold will increase relative to the normative threshold value derived via system II using expected utility threshold. This inclination for the higher diagnostic certainty may, in turn, explain undertreatment that is also documented in the current medical practice.

**Conclusions:**

We have developed the first dual processing model of medical decision-making that has potential to enrich the current medical decision-making field, which is still to the large extent dominated by expected utility theory. The model also provides a platform for reconciling two groups of competing dual processing theories (parallel competitive with default-interventionalist theories).

## Background

Dual processing theory is currently widely accepted as a dominant explanation of cognitive processes that characterizes human decision-making [1-9]. It assumes that cognitive processes are governed by so called system I (which is intuitive, automatic, fast, narrative, experiential and affect-based) and system II (which is analytical, slow, verbal, deliberative and logical) [1-10]. The vast majority of existing models of decision-making including expected-utility theory, prospect theory, and their variants assume a single system of human thought [11]. Recently, formal models for integrating system I with system II models have been developed [3,11]. One such attractive model-Dual System Model (DSM)- has been developed by Mukherjee [11]. Here, we extend Mukherjee’s DSM model to medical field (DSM-M) by linking it to the threshold concept of decision-making [12-15]. We also take into account decision regret, as an exemplar of affect or emotion that is involved in system I decision-making [2], and which is of particular relevance to medical decision-making [16-19]. Regret was also selected for use in our model because any “theory of choice that completely ignores feeling such as the pain of losses and the regret of mistakes is not only descriptively unrealistic but also might lead to prescriptions that do not maximize the utility of outcomes as they are actually experienced” [1,20].

As more than 30% of medical interventions are currently not appropriately applied, mostly as over - or- undertreatment [21-23], we illustrate how the DSM-M model may be used to explain the practice patterns seen in the current medical practice. Our DSM-M model is primarily an attempt to describe how medical decisions are made. As a descriptive model its validation will require comparing its outputs to actual choices made by patients and clinicians and their verbalized reactions to our model. We conclude the paper by providing some testable empirical predictions.

## Methods

### A dual system model

**Figure 1:**
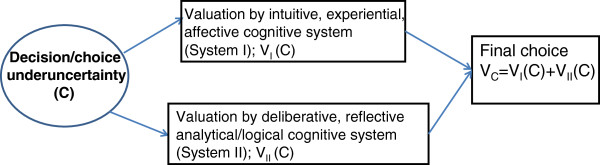
**Model of decision-making using dual processing cognitive processes (after Mukherjee [**[11]**]).**

Building on the previous empirical research, which has convincingly showed that human cognition is determined by both system I and system II processes [1,2,5,24,25]. Mukherjee recently developed a formal mathematical model, which assumes parallel functioning by both systems, while the final decision is a weighted combination of the valuations from both systems based on the value maximization paradigm (Figure 1) [11]. (NB. In this paper we employ terms system I and system II as popularized by Kahneman [1,2] although some authors prefer to talk about type 1 and 2 processing as it is almost certain that human cognition is not organized in distinctly separated physical systems [5,26,27]). 

Mukherjee’s dual system model (DSM) assumes that evaluation of risky choice (C) is formed by the combined input of system I and system II into a single value and can be formulated as follows:

(1) $$E\left(C\right)=\gamma V_{I}\left(C\right)+\left(1-\gamma \right)V_{\mathrm{II}}\left(C\right)=\gamma \frac{1}{n}\underset{i}{\sum }V_{I}\left(x_{i}\right)+\left(1-\gamma \right)k\underset{i}{\sum }p_{i}V_{\mathrm{II}}\left(x_{i}\right)$$

Where *C* represents a decision-making situation (“choice”), *n* - number of outcomes, *p*_*i*_ - probability of the *i*^*th*^ outcome, *x*_*i*_, of the selected choice. *V*_*I*_ represents valuation of decision under autonomous, intuitive, system I-based mode of decision-making and *V*_*II*_, which can be a utility function, represents valuation under a deliberative, rule-based, system II mode of decision-making. *k*-is a scaling constant, and *γ* [0 to 1] is the weight given to system I and can be interpreted as the relative extent of involvement of system I in the decision-making process [11]. System II is not split into two subsystems advocated by some [5], but is assumed to adhere to the rationality criteria of expected utility theory (EUT) as also advocated by modern decision science [11,28]. *γ* is assumed to be influenced by a number of processes that determine system I functioning. Mukherjee emphasized the following factors as the important determinants of system I functioning [11]: individual decision-making and thinking predispositions [ranging from expected utility theory (EUT) “maximizers” to system I driven “satisficing” with no regard to probabilities but with editing or selection of outcomes of interest] [29], affective nature of outcomes (the higher the affective nature of outcomes, the higher is *γ)* and framing and construing the decision-making task (decisions for the self will likely have higher *γ*, as well as decision problems that are contextualized and those requiring immediate resolution or are made under time pressure; the last four describe circumstances characteristic of medical decision-making). Easily available information, our previous experience, the way in which information is processed (verbatim vs. getting the “gist” of it) [30] as well as memory limitations [31] are also expected to affect *γ*. *γ* is, therefore, expected to be higher when information about probabilities and outcomes are ambiguous or not readily available, or when a very severe negative prior outcome is recalled [2,32,33]. On the other hand, when such data are available their joint evaluation by system II will reduce *γ*[11]. In general, the factors that define the process of system I can be classified under 4 major categories: a) affect, b) evolutionary hard-wired processes, responsible for automatic responses to potential danger in such a way that system I typically gives higher weight to potentially false positives than to false negatives (i.e. humans are cognitively more ready to wrongly accept the signal of potential harms than one that carries the potential of benefit), (c) over-learned processes from system II that have been relegated to system I (such as the effect of intensive training resulting in the use of heuristics, or “rules of thumb” or practice guidelines as one of the effort-saving cognitive strategies. NB although guidelines may be the products of analytic system II processes their applications tends to be a system I process.), and (d) the effects of tacit learning [5].

Mukherjee’s DSM model draws upon empirical evidence demonstrating that decision-makers in an affect-rich context are generally sensitive only to the presence or absence of stimuli, while in affect-poor contexts they rely on system II to assess the magnitude of stimuli (and probabilities) [24]. Hence, the salient feature of the model is that that system I recognizes outcomes only as being possible or, not. Every outcome that remains under consideration gets equal weight in system I. On the other hand, system II recognizes probabilities linearly without distortions, according to the expected utility paradigm.

As a result, dual valuation processing often generates instances where subjective valuations are greater at lower stimulus magnitudes (i.e. when decision-making relies on feeling, or evolutionary hard-wired processes such as when the signal may present danger) while rational calculation produces greater value at high magnitudes [11]. DSM is capable of explaining a number of the phenomena that characterize human decision-making such as a) violation of nontransparent stochastic dominance, b) fourfold pattern of risk attitude, c) ambiguity aversion, d) common consequences effect, e) common ratio effect, f) isolation effect, g) and coalescing and event-splitting effect [11].

Under the realistic assumption that outcomes are positive (i.e., utilities >0, which is particularly applicable to medical setting) and power value functions, $V_{I}\left(x\right)=x^{m_{I}}$, and $V_{\mathrm{II}}\left(x\right)=x\;$ for system I and system II, respectively, DSM can be re-written as:

(2) $$V\left(C\right)=\gamma \frac{1}{n}\underset{i}{\sum }x_{i}^{m_{I}}+\left(1-\gamma \right)k\underset{i}{\sum }p_{i}x_{i}$$

where 0 < *m*_*I*_ ≤ 1 Note that $x_{i}^{m_{I}}$ satisfies risk aversion for gains and risk seeking for losses and that the term for system II *p*_*i*_*x*_*i*_ is linear without risk distortions.

As noted by Mukherjee [11], the estimation of the parameters in Equation 2) is a measurement exercise, which needs to be evaluated in the future empirical research. Consequently, the functions *V*_*II*_*(x) and V*_*I*_*(x)* could be changed, depending on the decision-making setting and decision-maker’s goals. Similarly, parameter m may not be the same for all outcomes.

### Modification of DSM for medical decision-making

**Figure 2:**
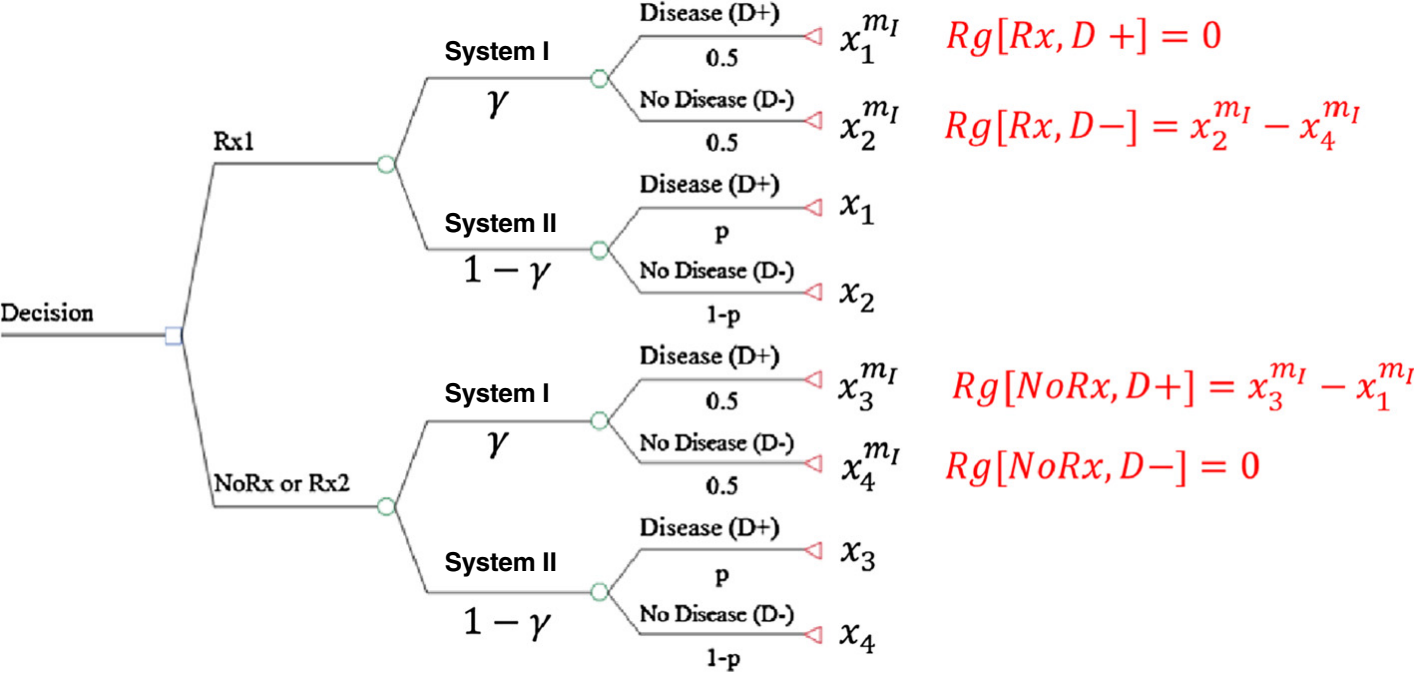
**Dual processing model of decision-making as applied to a clinical dilemma whether to treat (Rx) the patient with disease (D+) or not.** The patient may or may not have a disease (probability *p*). Regret is assumed to operate at the level of system I only. (Note that competing treatment alternative may include Rx or NoRx). Rg- regret.

We will consider a typical situation in clinical decision-making where a doctor has to choose treatment (Rx) vs. no treatment (NoRx) for disease (D) which is present with the probability p. [Note than NoRx represents a competing treatment alternative and may include a different treatment (Rx2)] [12,34]. Each decision results in outcomes that have a certain value, x_i_. The model is shown in the Figure 2. As noted above, the system I recognizes outcomes only as being possible (or not), and is thus insensitive to exact probabilities. Every outcome with non-zero probability gets equal weight in system I. Hence, in a two-alternative choice, each probability is equal to 0.5 under system I. System II recognizes probabilities without distortions, as would be expected according to EUT. 

We posit that among the emotions that can influence valuation of outcomes in system I processing, regret plays an important role [1,2], while system II processes are dominated by rational, analytical deliberations according to EUT [11]. We can define regret (Rg) as the difference (loss) in the utilities of the outcome of the action taken and that of the action we should have taken, in retrospect [16-19,35] but operating at the system I level only (see Figure 2).

Hence, we have the following value functions (see Additional file 1: Appendix for detailed derivation):

(3) $$\begin{array}{c} V_{I}\left(Rx,D+\right)=Rg\left[Rx,D+\right]=0;\\ V_{I}\left(NoRx,D+\right)=Rg\left[NoRx,D+\right]=x_{3}^{m_{I}}-x_{1}^{m_{I}};\\ V_{\mathrm{II}}\left(Rx,\;D+\right)=x_{1}\;;\\ V_{\mathrm{II}}\left(NoRx,\;D+\right)=x_{3}; \end{array}\;\begin{array}{c} V_{I}\left(Rx,D-\right)=Rg\left[Rx,D-\right]=x_{2}^{m_{I}}-x_{4}^{m_{I}}\\ V_{I}\left(NoRx,\;D-\right)=Rg\left[NoRx,\;D-\right]=0;\\ V_{\mathrm{II}}\left(Rx,\;D-\right)=x_{2};\\ V_{\mathrm{II}}\left(NoRx,\;D-\right)=x_{4}\text{;} \end{array}$$

Overall valuation of decision to treat (Rx) is equal to:

(4) $$\begin{array}{l} V\left(Rx\right)=\frac{\gamma }{2}\left(V_{A}\left(Rx,D+\right)+V_{A}\left(Rx,\;D-\right)\right)+\left(1-\gamma \right)k(pV_{D}\left(Rx,D+\right)+\left(1-p\right)V_{D}\left(Rx,\;D-\right))\\ \;=\frac{\gamma }{2}\left(x_{2}^{m_{A}}-x_{4}^{m_{A}}\right)+\left(1-\gamma \right)k\left[px_{1}+\left(1-p\right)x_{2}\right] \end{array}$$

And

(5) $$\begin{array}{l} V\left(NoRx\right)=\frac{\gamma }{2}\left(V_{A}\left(NoRx,D+\right)+V_{A}\left(NoRx,\;D-\right)\right)+\left(1-\gamma \right)k\left(pV_{D}\left(NoRx,D+\right)+\left(1-p\right)V_{D}\left(NoRx,\;D-\right)\right)\\ \;=\frac{\gamma }{2}\left(x_{3}^{m_{A}}-x_{1}^{m_{A}}\right)+\left(1-\gamma \right)k\left[px_{3}\;+\left(1-p\right)x_{4}\right] \end{array}$$

The difference in the outcomes of treating and not treating patient with disease are equal to the net benefit of treatment (B) [13,14,36]; the difference in outcomes of not treating and treating those patients without disease is defined as net harms (H) [13,14,36]. Note that benefits and harms can be expressed in the various units (such as survival, mortality, morbidity, costs, etc.) and can be formulated both as utilities and disutilities [13,14,36]. As explained above, we further assume that valuation of net benefits and net harms by system I differs from system II. Hence, under system II, we replace net benefit and net harms using EUT definitions:$B_{\mathrm{II}}=x_{1}-x_{3}$ and net harms $H_{\mathrm{II}}=x_{4}-x_{2}$. Under system I, we define $B_{I}=x_{1}^{m_{I}}-x_{3}^{m_{I}}$, and $H_{I}=x_{4}^{m_{I}}-x_{2}^{m_{I}}$. Solving for *p* (the probability of disease at which we are indifferent between Rx and NoRx), we obtain: (Equation 3)

(6) $$\begin{array}{l} p_{t}=\;p=\frac{\left(1-\gamma \right)kH_{\mathrm{II}}-\frac{\gamma }{2}\left[B_{I}-H_{I}\right]}{\left(1-\gamma \right)k\left[B_{\mathrm{II}}+H_{\mathrm{II}}\right]}=\frac{1}{1+\frac{B_{\mathrm{II}}}{H_{\mathrm{II}}}}-\frac{\gamma }{2k\left(1-\gamma \right)}\frac{B_{I}-H_{I}}{B_{\mathrm{II}}+H_{\mathrm{II}}}\\ \;=\;\left(\frac{1}{1+\frac{B_{\mathrm{II}}}{H_{\mathrm{II}}}}\right)\left[1+\frac{\gamma }{2\left(1-\gamma \right)}\left(\frac{H_{I}}{H_{\mathrm{II}}}\right)\left(1-\frac{B_{I}}{H_{I}}\right)\right]=\;\left(p_{t}\left(EUT\right)\right)\left[1+\frac{\gamma }{2\left(1-\gamma \right)}\left(\frac{H_{I}}{H_{\mathrm{II}}}\right)\left(1-\frac{B_{I}}{H_{I}}\right)\right] \end{array}$$

This means that if the probability of disease is above *p*_*t*_ the decision-maker favors treatment; otherwise, a competing management alternative (such as “No Treatment”) represents the optimal treatment strategy. Note that k can be typically set at 1, as we do it here. Also note that the first part of equation is equivalent to the threshold expression described in EUT framework [13,14,36]; the second expression modifies system II’s EUT-based decision-making process in such a way that if benefits are experienced higher than harms, the threshold probability is always lower than EUT threshold. However, if a decision-maker experiences *H*_*I*_*>B*_*I*_, the threshold probability is always higher than the EUT threshold (see below for discussion in the context of medical example). Note that *γ* and the ratio $\frac{H_{I}}{H_{\mathrm{II}}}$ only contribute to the extent of magnitude the dual threshold is above or below the classic EUT threshold. That is, *γ* and the ratio $\frac{H_{I}}{H_{\mathrm{II}}}$ do not change the quality of relationship between dual threshold and EUT threshold: whether dual threshold will be above or below the EUT threshold depends only on a $\frac{B_{I}}{H_{I}}$ ratio.

It should be noted that the identical derivations can be obtained by applying the concept of expected regret (instead of EUT) [16-19,35]. Although it can be argued that regret is a powerful emotion influencing all cognitive processes (as so called, “cognitive emotion”) [37,38], and so it may function at level of both system I and system II [39], most authors recognize the affect value of regret [2,10]. Hence, we assumed that regret functions at system I level [2]. Therefore, in our model we restrict the influence of regret to system I. Incidentally, our Equation 3) can also be derived from the general Mukherjee’s DSM model even if regret is not specifically invoked [11].

Although Equation 3) implies exact calculations, it should not be understood as one that provides precise mathematical account of human decision-making. Rather, it should be considered more as a semi-quantitative or qualitative description of the way physicians may make their decisions. First, this is because system I does not perform exact calculations, but rather relies on “gist” [30,31] for assessment of benefits and harms in more qualitative manner. The mechanism depends on associations, emotions (so called, “risk as feelings” estimates [10]), as well as memory, and experience [2,5,8,31]. In this sense, the second part of Equation 3) that relies on system I can be understood as the qualitative modifier (“weight”), which, depending on the system I’s estimates of benefits and harms increases or decreases the first part of equation (which is dependent on system’s II precise usage of evidence for benefits and harms). Second, the threshold probability itself should be considered as an “action threshold”- at some point, a physician decides whether to administer treatment or not. Typically, she contrasts the estimated probability of disease against the threshold and acts: if the probability of disease is above the “action threshold”, the physician administers the treatment; if it is below, she decides not to give treatment. So, one way to interpret Equation 3) is to consider physician’s estimate of “gist” of the action threshold: if in her estimation, overall benefits of treatment outweigh harms, and she considers that it is “likely” that the probability of disease is above the threshold probability, then she would act and administer treatment. If the physician assesses that it is “unlikely” that the probability disease is above the “action threshold”, then she would not prescribe the treatment.

### The behavior of DSM-M model

The exact cognitive mechanisms that underlie dual system processes are not fully elucidated. As discussed throughout this paper, many factors affect dual processes reasoning leading to suggestions that these processes should be grouped according to the prevailing mechanisms [27]. Focusing on each of these processes may lead to specific theoretical proposals. Our goal in this paper is to provide overarching cognitive architecture encompassing general features of the majority existing theoretical concepts, while at the same time concentrating on specifics of medical decision-making. In general, dual processing theories [27] fall into two main groups [27,40] parallel competitive theories and default-interventionalist theories. The parallel-competitive theories assume that system I and II processes proceed in parallel, each competing for control of the response [27]. If there is a conflict, it is not clear which mechanism is invoked to resolve the conflict [27]. On the other hand, default-interventionist theories postulate that system I generates a rapid and intuitive default response, which may or may not be intervened upon by subsequent slow and deliberative processed of system II [2,5,27]. This can be further operationalized via several general mechanisms that have been proposed in the literature:

1) Mukherjee’s additive model as described above [11]. It can be categorized as a variant of parallel-competitive theory as it assumes that system I and II processes proceed in parallel, but does include parameter *γ*, which can trigger greater or smaller activation of system I. Mukherjee’s model, however, does not explicitly model the choices in terms of categorical decisions (i.e. accept vs. do not accept a given hypothesis), which is a fundamental feature of dual-processing models [27].

2) System I and system II operate on a continuum [41], but in such a way that system I never sleeps [2]. A final decision depends on the activation of both systems I and II [2]. It has been estimated that about 40-50% of decisions are determined by habits (i.e. by system I) [42]. This is also a variation of parallel-competitive theory; it should be noted that latest literature is moving away from this model [5,27].

3) The final decision appears to depend both on the system I and system II in such a way that system I is the first to suggest an answer and system II endorses it [2]. In doing so, system II can exert the full control over system I (such as when it relies on the EUT modeling) or completely fail to oversee functioning of system I (e.g., because of its ignorance or laziness) [2]. Therefore, according to this model, decisions are either made by system I (default) or system II (which may or may not intervene). This is a default-interventionalist model.

4) The variation of the model #3 is the so called “toggle model”, which proposes that decision-maker constantly uses cognitive processes that oscillate between the two systems (toggle) [6,7,9]. This is a variant of default-interventionalist model.

Note that *γ* is continuous in our model, but it can be made categorical [0,1] if the “toggle” theory is considered to be the correct one. In this case, a logical switch can be introduced in the decision tree to allow toggling between the two systems. Most importantly, by linking Mukherjee’s additive model with the threshold model, we provide the architecture for reconciling parallel competitive theories with default-interventionalist theories. We do it by making explicit that decisions are categorical (via threshold) at certain degree of cognitive effort (modeled via *γ*) parameter [27]. That is, the key question is what processes determine acceptance or rejection of a particular (diagnostic) hypothesis. Our model shows that this can occur if we maintain parallel-competing architecture of Mukherjee’s additive model but assume a switch, yes or no answer, whether to accept or reject a given hypothesis (*at the threshold*). It is evaluation of the (diagnostic) event with respect to the threshold that serves as the final output of our decision-making and reasoning processes. As our model shows, this depends on assumption of parallel working of both system I and system II, *and* the switch in control of one system over another according to default-interventionalist hypothesis. Note that depending on activation of *γ* parameter and assessment of benefits (gains) and harms (losses) the control can be exerted by either system: sometimes it will be the intuitive system that it will exert the control and our action will take the form “feeling of rightness” [43]; sometimes, it will be system II that it will prevail and drive our decisions. Thus, we succeed in uniting parallel competitive with default-interventionalist models by linking Mukherjee’s additive model with the threshold model for decision-making.

As discussed above, many factors can activate the switch such as the presence or absence of empirical, quantitative data, the context of decision making (e.g. affect poor or rich), the decision maker’s expertise and experience, etc. In addition, extensive psychological research has demonstrated that people often use a simple heuristic, which is based on the prominent numbers as powers of 10 (e.g., 1,2,5,10,20,50,100,200 etc.) [44]. That is, although system I does not perform the exact calculations, it still does assess “gist” of relative benefits and harms, and likely does so according to “1/10 aspiration level” [44] (rounded to the closest number) in such a way that the estimates of benefits/harms ratio change by 1,2,5, 10, etc. orders of magnitude. Therefore, in this section we consider several prototypical situations: 1) when γ = 0, 0.5, or 1; 2) when B_II_> > H_II_, B_II_ = H_II_ and B_II_ < <H_II_; and 3) when regret of omission (B_I_) < < regret of commission (H_I_), B_I_ = H_I_, or B_I_> > H_I_

First, note that *γ=0*, when the numerator of the left fraction in the Equation 6 ( Additional file 1: Appendix) is zero, i.e., when $pB_{\mathrm{II}}-\left(1-p\right)H_{\mathrm{II}}=0$, or solving for *p*, we obtain $p=\frac{1}{1+\frac{B_{\mathrm{II}}}{H_{\mathrm{II}}}}$, which is exactly the value of the EUT threshold for the probability at which the expected utilities of the two options are the same. This will correspond to model #3 above, in which system II exerts full control over decision-making. Therefore, when γ = 0, we have the classic EUT and therapeutic threshold model. In this case, regret does not affect the EUT benefits and harms, and $p_{t}=\;\frac{H_{\mathrm{II}}}{H_{\mathrm{II}}+B_{\mathrm{II}}}=\frac{1}{1+\frac{B_{\mathrm{II}}}{H_{\mathrm{II}}}}$. If B_II_> > H_II_, p_t_ approaches zero and a decision-maker will recommend treatment to virtually everyone. On the other hand, if B_II_ = H_II_, p_t_ equals 0.5 and she might recommend treatment if the disease is as likely as not. Finally, if B_II_ < < H_II_, p_t_ approaches 1.0, and the decision-maker is expected to recommend treatment only if she is absolutely certain in diagnosis.

At the other extreme, if γ = 1, we have the pure system I model (corresponding to model #3 above, which solely relies on system I processes). Note the value of *γ*=1, when the denominator of the second fraction in Equation 6 ( Additional file 1: Appendix) equals one, or when the expression $H_{I}-B_{I}=0$, i.e., when *B*_*I*_*=H*_*I*_. Under these conditions, it is fairly obvious that the system I assessments become irrelevant if the perceived net benefit of the treatment is equal to the perceived net harm. When *γ*=1, regret avoidance becomes the key motivator, not EUT’s benefits and harms. Note that in system I p is not related to *γ* in terms of the valuation (Equation 1). Under these circumstances only decision-making under system I operate and the analytical processes of system II are suppressed (Equation 1) as seen in those decision-makers who tend to follow intuition only, or are extremely affected by their past experiences without considering new facts on the ground. That is, differences in probability do not play any role in such decisions, because a person who only uses system I doesn’t consider probability as a factor.

Finally, if γ = 0.5, the decision maker is motivated by EUT and by regret avoidance (model #2 listed above). In this case, the benefits (B_II_), harms (H_II_), regrets of omission (B_I_) and commission (H_I_) are all active players. These three cases are presented in Table 1 (see Additional file 2) which shows threshold probabilities for γ = 0.5 and objective data indicating a high benefit/harms ratio (${\text{B}}_{\text{II}}/{\text{H}}_{\text{II}}=10$). Also shown is how the threshold probability depends on individual risk perception. If H_I_> > H_I_, it magnifies effect of B_I_/H_I_ (see Equation 3), which results in extreme behavior in sense of increasing likelihood that such a person will either always accept (as p_t_<0) or reject treatment (as p_t_>1). For H_I_ < <H_II_, the impact on the way system I processes benefits and harms is not that pronounced and influences the EUT threshold to much smaller extent.

## Results

### Illustrative medical examples

Clinical examples abound to illustrate applicability of our model. To illustrate the salient points of our model, we chose two prototypical examples where there is close trade-offs between treatments’ benefits and harms.

### Example #1: treatment of pulmonary embolism

Pulmonary embolism (PE) (blood clot in the lungs) is an important clinical problem that can lead to significant morbidity and death [45]. Even though many diagnostic imaging tests exist to aid in the accurate diagnosis of PE, the tests are often inconclusive, and physicians are left to face the decision whether to treat patient for presumptive PE, or attribute the patient’s clinical presentation (such as shortness of breath and/or chest pain) to other possible etiologies. There exists an effective treatment for a PE, which consists of the administration of 2 anticoagulants (blood thinners): heparin followed by oral anticoagulants such as warfarin [46,47]. Heparin (unfractionated or low-molecular weight heparins) are highly effective treatments associated with relative risk reduction of death from PE by 70-90% in comparison to no treatment [46,47]. This converts into the absolute death reduction as: net benefits, *B*_*II*_=17.5% *to* 22.5% (calculated as 25% morality without heparin minus 7.5% to 2.5% with heparin) [17,18,46,47]. However, these drugs are also associated with a significant risk of life-threatening bleeding; net harms range from *H*_*II*_=0.037% (a typical scenario) to 5% (a worst-case scenario) depending on the patients’ other comorbid conditions [17,18,47,48]. Thus, net benefits/net harms range from 60.8 (22.5/0.037) (best case) to 3.5 (17.5/5)(worst case scenario). If we apply a classic EUT threshold [13,14,36], which relies solely on system II processes, we observe that the probability of pulmonary embolism above which the physician should administer anticoagulants ranges from 1.6% $=1/\left(1+60.8\right)$ (best case) to 22.2% $1/\left(1+3.5\right)$(worst case scenario). However, ample clinical experience has demonstrated that few clinicians would consider prescribing anticoagulants at such low probability of PE [18]. In fact, most experts in the field recommend giving anticoagulants when probability of PE exceeds 95% [49-51]. We have previously suggested that this is because regret associated with administering unnecessary and potentially harmful treatments under these circumstances likely outweighs regret associated with failing to administer potentially beneficial anticoagulants [17-19]. We now show how this argument can be made in the context of dual processing theory. Indeed, some physicians may feel that the risk of bleeding may be much higher, particularly in case of a patient who recently experienced major hemorrhage. The physician may not have data readily available to adjust her EUT, system II-based calculations. Rather, she employs the system I-based reasoning, globally assessing the benefits and harms of treatments under her disposal. Importantly, these are personal, intuitive, affect-based, subjective judgments of the values of outcomes that are influenced by memory limitations and recent experiences and that may not be objectively based on the external evidence [2,30-33]. In addition, it is well documented that the physicians’ recent experience leads to a type of bias, known as primacy effect, that is governed by system I [2,33]. If the last patient with PE whom the physician took care of had severe bleeding, system I may be primed in such a way that it will likely conclude that harms outweigh benefits. In our case of PE, if her reasoning is dominated by system I (operating, say, at *γ* level of 0.77 according to model #2 listed above, see Section “The behavior of DSM-M model”) in a such way that the physician concludes that if harms is larger than benefits by 10%, then the threshold probability above which she will treat her patient suspected of PE exceeds 95% [as easily demonstrated after plugging in the benefits/harms values in Equation 3) $p_{t}\left(\text{dual}\right)=.222-\left(.77\right)/\left(2*.23\right)*\left(-.10/.225\right)=0.966=96.6\%\;\text{for k}=1$. Note that this calculation describes circumstances under which the physician would adhere to the contemporary practice guidelines i.e. to prescribe anticoagulants when PE exceeds 95% [49-51]. It should be further noted that if *γ* value is only slightly higher (≥0.78), the physician will require the absolute certainty to act (i.e. the threshold ≥1).

**Figure 3:**
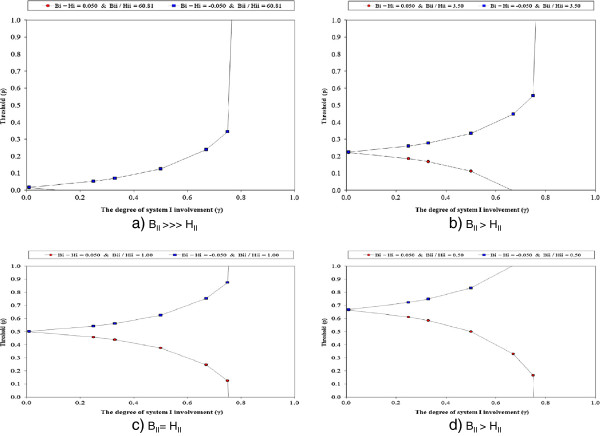
**Dual decision threshold (p**_**t**_**) as the function of relative involvement of systems I and II in dual process model of medical decision making: a) objective data show very high benefit/harms ratio (B**_**II**_**> > H**_**II**_**), b) moderately high benefit/harms ratio (B**_**II**_ **> H**_**II**_**), c) B**_**II**_ **= H**_**II**_**, d) B**_**II**_ **< H**_**II**_**.** The intercept at y axis threshold probability. The graph shows how the threshold is affected by the extent of system I involvement (*γ*) and whether system I perceives that benefits is greater than harms [by 5% in this example](red lines, circles) or that harms outweigh benefits[by 5%] (blue line, squares). Decision-maker accepts treatment if the probability of disease exceeds the threshold; otherwise, treatment would not be acceptable (see text for details).

DSM offers an account of the opposite behavior as well i.e. the threshold based on global evaluation using both system I and system II can also be lower than the EUT threshold (if *B*_*I*_*>H*_*I*_ additive, model #1, Equation 3). For example, the physician may trivialize the risks of treatment and believe that the benefits are much higher than the treatment harms. As a result, the threshold above which she commits to treatment drops below EUT threshold (as predicted by Equation 3). Figure 3 shows how the decision threshold (p_t_) is affected by the relative involvement of systems I and II in dual process model of medical decision-making in the “best” (${\text{B}}_{\text{II}}/{\text{H}}_{\text{II}}\;=\;60.8$) and “worst case” scenario (${\text{B}}_{\text{II}}/{\text{H}}_{\text{II}}=3.5$) for treatment of PE and when system I valuation of benefits is greater than harms or when harms are perceived to outweigh benefits. It can be seen that when objective data indicate that benefits considerably outweigh harms (${\text{B}}_{\text{II}}>>>{\text{H}}_{\text{II}}$) (as when ${\text{B}}_{\text{II}}/{\text{H}}_{\text{II}}=60.8$), then as long as system I values benefits as being greater than harms, the threshold dramatically drops to zero indicating that the extent of system I involvement (i.e. *γ* value) in decision-making is of little consequence. However, if system I clashes with objective data, then the probability of PE above which the decision-maker is prepared to treat, dramatically increases (Figure 3a). Similarly, in all other circumstances (when B_II_ > H_II,_ B_II_ ~ H_II_, B_II_ < H_II_), the threshold probability is significantly affected by involvement of system I (Figures 3b–3d).

### Example #2: treatment of acute leukemia

Acute myeloid leukemia (AML) is a life-threatening disease, which, depending on the aggressiveness of disease can be cured in the substantial minority of patients. To achieve a cure, patients are typically given induction chemotherapy to bring the disease into remission, after which another form of intensive therapy - so called, consolidation treatment - is given. To achieve a cure in patients with more aggressive course of disease such as those classified as intermediate- and poor-risk AML based on cytogenetic features of disease, allogeneic stem cell transplant (alloSCT) is recommended [52]. However, the cure is not without price- many patients given alloSCT as a consolidation therapy die due to treatment. A decision dilemma faced by a physician is whether to recommend alloSCT, or alternative treatment, such as chemotherapy or autologous SCT, which has lower cure rate but less treatment-related mortality. In intermediate-risk AML, for example, credible evidence shows that, compared with chemotherapy allogeneic alloSCT result in better leukemia-free survival (LFS) by at least 12% at 4 years (LFS with alloSCT =53% vs 41% with chemotherapy/auto SCT) [53]. Treatment-related mortality is much higher with alloSCT by 16%, on average (19% with alloSCT vs. 3% with chemotherapy/autoSCT) [53]. This means that based on objective data, and using rational EUT model, we should recommend alloSCT for any probability of AML relapse ≥57.1% $\left(\text{threshold}=1/\left(1+0.12/0.16\right)=0.571\right)$. Therefore, treatment benefits and harms are, on average, very close. Because of this, the driving force to recommend alloSCT is the physician’s estimates of the patient’s tolerability of alloSCT: if she assess that the patient will not be able to tolerate alloSCT, the physician will not recommend transplant. Conversely, if she thinks that the patient will be able to tolerate allo SCT, the physician will recommend it. Although there are objective criteria to evaluate a patient’s eligibility for transplant, the assessment to the large extent depends on physicians’ judgment and experience [54]. That is, the assessment of patient’s eligibility for transplant depends both on the objective data on benefits and harms (system II ingredients) and intuitive, gist type of judgment (characteristics of system I). As discussed above, system I does not conduct the precise calculations. Rather, it relies on “gist” or on simple heuristics such as those that are based on powers of 10 (e.g., 1,2,5,10,20, etc.) [42]. The physician, therefore, adjusts the threshold above or below based on her intuitive calculations. For instance, it is often the case that the physician whose patient recently died during the transplant is more reluctant to recommend the procedure even to those patients who, otherwise, seems fit for it. In doing so, the physician in fact modifies her/his dual system threshold upwards. In our example, let’s assume that the physician judges that the harms of alloSCT for a given patient is twice as large as reported in the studies where patients were carefully selected for transplant [52]. That, in our case, would mean that mortality due to alloSCT is 32% (instead of 16%). We can now plug these numbers in Equation 3) (B_II_ = 0.12, H_II_ = 0.16, B_I_ = 0.12, H_I_ = 0.32).

Note that the physician can make this judgment at various level of activation of system I. If the decision is predominantly driven by system I judgment then our physician’s threshold according to Equation 3) is greater than 100% for all circumstances in which *γ* value exceeds 55%. That means that under these circumstances of system I activation, the physician will never recommend transplant. The opposite can occur for those physicians whose experience is not affected by poor patients’ outcomes. Under such circumstances, the physician may judge the patient to be in such a good condition that she may re-adjust the reported treatment-related transplant risk to be as half of those observed risks in the published clinical studies (i.e. 8%). The new numbers required to determine the threshold according to Equation 3 are: B_II_ = 0.12, H_II_ = 0.16, B_I_ = 0.12, H_I_ = 0.08. If the physician relies excessively on system I, as often seen in busy clinics where decisions are routinely made on “automatic pilot”, the dual threshold drops to zero (for all *γ* >89%). That means, that the physician will recommend alloSCT to all her/his patients under these circumstances.

As discussed above, we provide the precise calculations only to illustrate the logic of decision-making. The process should be understood more along semi-quantitative or qualitative description of clinical decision-making. Although currently the Equation 3) allows entry of almost any value for benefit and harms, it is probably the case that benefit and harms as perceived by system I are based on “1/10 aspirational level” [44], so that only values of 1,2,5,10, 20 etc. should be allowed. This is, however, empirical question that should be answered in further experimental testing; therefore, at this time, we decided not to provide the exact boundaries of the values for benefit and harms that can be entered in Equation 3 (see Discussion). Note also that these calculations are decision-maker specific, and although we illustrate them from the perspective of the physician, the same approach applies to the patient, who ultimately has to agree –based on her own dual cognitive processing- on the suggested course of treatment actions.

## Discussion

Models of medical decision-making belong to two general classes-descriptive and prescriptive. The former, which the DSM-M exemplifies, attempt to explain why decision makers take or might take certain actions when presented with challenging decision problems abundant in contemporary medicine. The latter, exemplified by the normative therapeutic threshold models [13,14] prescribe the choice options that a rational decision maker should take. We have defined the first formal dual-process theory of medical decision-making by taking into consideration the deliberative and the experiential aspects that encompass many of the critical decisions physicians face in practice. Mathematically, our model represents an extension of Mukharjee’s additive Dual System Model [11] to the clinical situation where a physician faces frequent dilemmas: whether to treat the patient who may or may not have the disease, or choose one treatment over another for prevention of disease that is yet to occur. Our model is unique in that incorporates an exemplar of strong emotion, decision regret, as one of the important components of system I functioning. We focused on regret because previous research has shown that people often violate EUT prescribed choice options in an effort to minimize anticipated regret [1,2,20]. Although we use the more common psychological term “regret,” the concept is analogous to Feinstein’s term “chagrin” [55]. In fact, explicit consideration of post-choice regret in decision making has been considered an essential element in any serious theory of choice and certainly dominates many clinical decisions [1,2,20]. We also reformulated the original model using the threshold concept- a fundamental approach in medical decision-making [13,14,36]. The threshold concept represents a linchpin between evidence (which presents on the continuum of credibility) and decision-making, which is a categorical exercise (as choice options are either selected or not) [13,14,36]. Using an example such as pulmonary embolism, we have shown how the extended model can explain deviations from outcomes predicted by EUT, and account for the variation in management of pulmonary embolism [45]. In general, it is possible that the huge practice variation well documented in contemporary medicine [56-61], can be, in part, due to individual differences in subjective judgments of disease prevalence and “thresholds” at which physicians act. [17,18,62]. This may be because quantitative interpretations of qualitative descriptors such as rarely, unlikely, possible, or likely [63] differ markedly among individuals and hence “gist” representations of a given clinical situation can vary widely among different physicians [30]. We are, of course, aware that many other factors contribute to variation in patient care including the structure of local care organizations, the availability of medical technologies, financial incentives etc [60]. Our intent in this article is to highlight, yet another important factor- individual differences in risk assessment as shaped by different mechanisms operating within a dual process model of human cognitive functioning [5].

**Figure 4:**
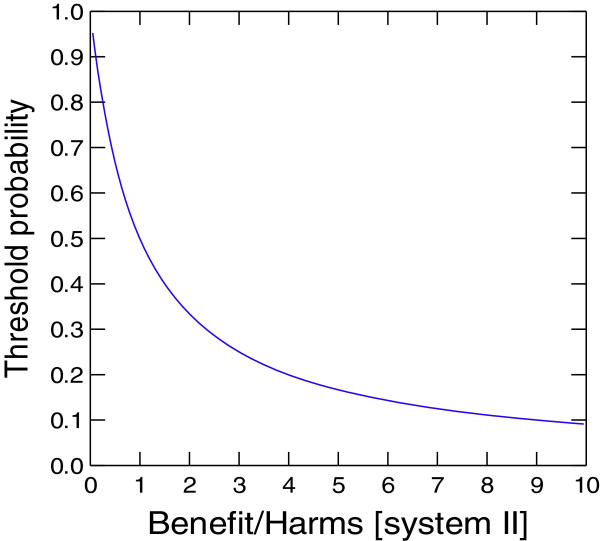
**Dual Decision Threshold Model.** Classic, expected utility threshold probability as a function of benefit/harms ratio as derived by system II, EUT (expected utility threshold) (solid line). The treatment should be given if the probability of disease is above the threshold, otherwise should be withheld. Note that if system I perceives that harms are higher than system I benefits (B_I_ < H_I_), the threshold probability is always higher than classic EUT (dotted line). However, if B_I_ > H_I,_ the threshold probability is always lower than the EUT threshold (dashed line) (see text for details).

There are many theories of decision-making [64]. Most assume a single system of human reasoning [11]. Nevertheless, all major theories of choice agree that rational decision-making requires integrations of benefits (gains) and harms (losses). EUT vs. non-EUT theories of decision-making differ in how benefits and harms should be integrated in a given decision task. To date, dual processing theory provides the most compelling explanation how both intuitive and rational cognitive processes integrate information on benefits and harms and provide not only descriptive assessments of decision-making, but possibly may lead to insights that improve the way decisions are made. Figures 3 &4 illustrate how dual decision threshold (shown on the Y axis) for deciding between two possible courses of action can be influenced by the degree of system I involvement. As discussed above and mathematically captured in Equation 3, the clinical action such as treat versus no treat is best explained by relating benefit and harms of proposed therapeutic interventions to the threshold probability: if the estimated probability of disease is greater than the threshold probability, then the decision-maker is inclined to give treatment; if the probability of disease is below the threshold, then the treatment is withheld. Figure 4 shows a dramatic drop in the decision threshold as a function of the ratio between benefit and harms, which is derived from empirically obtained evidence. When these data are solely relied on by system II, the rational course of action consists of administering treatment as long as the probability of disease is above the threshold regardless how low the threshold probability drops [13,14,36] (which in case of the treatment of a patient with pulmonary embolism can be as low as 1.6%!). Paradoxically, if we were to adopt this – presumably most rational-approach to the practice of medicine, we would likely see a further explosion of inappropriate and wasteful use of health care resources [18,21]. This is because in today’s practice, benefits of approved treatments vastly outweigh their harms, and as a result threshold probability values is predictably very low for the majority of health care interventions employed in the contemporary clinical practice [18]. System I, however, does offer a means of mitigation. The correction of the thresholds - our action whether we are comfortable treating at higher or lower probability than the thresholds obtained via usage of system II – depends on the extent of involvement of system I in decision-making. If system I perceives that harms are higher than system I benefits, the threshold probability is always higher than classic EUT threshold. However, if *B*_*I*_*>H*_*I*_, the threshold probability is always lower than the EUT threshold (Figure 4). This is particularly evident in clinical practice when physicians attempt to tailor evidence based on the results of the research study, which generates the “group averages”, to individual patients who often differ in important ways from patients enrolled in the research studies (e.g., these patients may be older, have comorbid conditions, might be using multiple medications, etc.) [65]. It is under these circumstances that system I affects our judgments and can give rise to different decisions from those based solely on system II. Note, however, that although system I does assess benefits and harms, it likely does so via”gist” representation and not necessarily by employing the exact numerical values as system II does [30]. System I is also affected by emotions, as illustrated in the case where experts panels of the governments of many countries recommended H1N1 influenza vaccination, but where inoculation was refused by the majority of patients [66,67]. 

It is interesting to examine circumstances under which we always treat (*p*_*t*_ *≤ 0)* or never treats (*p*_*t*_ *≥ 1).* Equation 1 ( Additional file 2: Table S1) shows that when objective evidence indicates that benefits outweigh harms, and when this is further augmented by the decision-maker’s risk attitude in such a way that it magnifies system I’s valuation of benefits and harms, then we can expect to continue to witness further overtreatment in clinical practice (as p_t_ drops to zero) [65]. However, when the decision-maker perceives the benefits smaller than harms, then the threshold increases; consequently, the decision-maker will require higher diagnostic certainty before acting (Figure 3 & Figure 4). This may occur during extrapolation of research results from the group averages to individual patients, when empirical evidence about B_II_ and H_II_ is considered to be unreliable, when the decision-maker is risk averse, or when his or her cognitive processes are biased through the distorting effects of recent experience, memory limitations or other forms of biases well described in the literature [2,31,33]. This discussion illustrates how the “rationality of action” may require a re-definition, one encompassing both the formal principles of probability theory and human intuitions about good decisions [5,68]. Our goal here is not to demonstrate that one approach is conclusively superior to the other- we are merely outlining the differences in the current physicians’ behavior from the perspective of dual processing theory.

Despite the growing recognition of the importance of dual processing for decision-making [2,5], a few formal models have been developed to try to capture the essence of the way we make decisions. Because different authors focus on different aspects of a multitude of decision-making processes, Evans has recently pointed out that there are many dual processing theories [27] which fall into two main groups [27,40] parallel competitive theories and default-interventionalist theories. While the exact accounts of cognitive processes between these two groups of theories differ [27], as discussed above *(Section The behavior of DSM-M Model)*, we, for the first, time provide a platform, albeit the theoretical one, for reconciling parallel competitive theories with default-interventionalist theories.

Nevertheless, our main goal is to define a theoretical model for medical decision-making; such a model may enable creation of new theoretical frameworks for future empirical research. Future research, obviously, involves extension of the model described herein to more complex clinical situations beyond relatively simple two-alternative situation, even if the latter is frequently encountered in practice. Particularly interesting will be the extension of our dual processing model to include the use of diagnostic tests as the number of new diagnostic technologies continues to explode. Finally, and most importantly, the model presented here needs empirical verification. This limitation is not unique to our model, however, and this criticism can be leveled against most current medical decision-making models, which are rarely, if ever, subjected to empirical verification.

Our model heavily relies on Mukherjee’s model [11], and is accurate to the extent his additive dual processing model is correct (Figure 1, Equations 1 & 2). Also, note that we have extended Mukherjee’s DSM model by omitting his scaling constant *k* and using general utility expressions, rather than a single parameter monotonic power function. As discussed above, many factors can activate the switch of system II. In fact, Kahneman warns [2] that “because you have little direct knowledge what goes on in your mind, you will never know that you might have made a different judgment or reached a different decision under very slightly different circumstances”. This implies that the multiple factors affecting the gamma parameter cannot be directly modeled. A possible solution –and area for future research building on the psychological “fuzzy trace theory” [30]-would be to employ a fuzzy logic model to assess the values of *γ* (and threshold) as a function of multiple fuzzy inputs [69].

The complexity described here notwithstanding, we believe that the empirical verification of our current dual processing model is feasible. Even without direct modeling of all factors affecting *γ* parameter, our model generates empirically falsifiable qualitative predictions as it clearly identifies circumstances under which the *decision threshold* is increased or decreased as a function of activation of system I (*γ* parameter). Using simulation to imitate the various real-life decision-making scenarios [70] offers most logical avenue toward the first empirical testing of our model.

Our model also holds promise in medical education. As highlighted in Introduction, modern knowledge of cognition has taught us that most people, including physicians process information using both system I (fast, intuitive) and system II (slow, deliberative) reasoning at different times but few investigators have examined how to teach physicians to integrate both modes of reasoning in arriving at therapeutic strategies. On the diagnostic side, many investigators [6,71] have examined clinical reasoning and proposed how experienced physicians move between system I and system II, although most early papers used different terminology. The integration of system I and system II in therapeutic decision making in medicine has been less well examined. A number of investigators have proposed approaches to using and teaching system II reasoning, including the use of decision models [71]. Although this is taught in some schools it has not yet taken medical education by storm [71]. In the field of economic analysis Mukerjee has proposed a theoretical means of combining system I and system II reasoning. In this paper, we build on Mukurjee’s work and show how the integration of system I and system II therapeutic reasoning can form a basis for teaching students and experienced physicians to recognize and integrate system I and system II reasoning. Our model uniquely captures most salient features of (medical) decision-making, which can be effectively employed for didactic purposes. It is believed that by recognizing separate roles of system II and the influence of system I mechanisms on the way we make decisions, we can be in a better position to harness both types of processes toward better practice of making clinical decisions [2,9].

## Conclusion

We hope that our model will stimulate new lines of empirical and theoretical work in medical decision-making. In summary, we have described the first dual processing model of medical decision-making, which has potential to enrich the current medical decision-making field dominated by expected utility theory.

## Competing interests

The authors declare that they have no competing interests.

## Authors' contributions

BD had an idea for the study. BD & IH jointly developed the model. IH solved the model. BD and IH performed the analyses. JB and AT performed additional analyses. SGP analyzed the performance of dual processing model and provided an additional intellectual input. BD wrote the first draft. All authors read and approved the final manuscript.

## Pre-publication history

The pre-publication history for this paper can be accessed here:

http://www.biomedcentral.com/1472-6947/12/94/prepub

## Supplementary Material

Additional file 1 Appendix: Derivation of DSM-M equation. Click here for file

Additional file 2 ** Table S1.** Evaluation of Behavior of Dual Processing Model for Medical Decision-Making (DSM -M). Threshold probability as a function of individual risk perception. Click here for file
