# Supplementary material for: Dual processing model of medical decision-making
Source: BMC Med Inform Decis Mak. 2012 Sep 3;12:94. doi: 10.1186/1472-6947-12-94 (PMC3471048; doi:10.1186/1472-6947-12-94)
Supplement: Additional file 1 — Appendix: Derivation of DSM-M equation. [file 1472-6947-12-94-S1.doc]

**Appendix**

**Derivation of DSM-M equation:**

As shown in Fig 2, we have the following value functions:

| 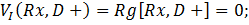  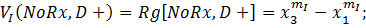  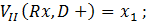  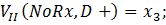 | 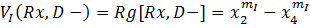  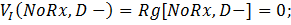  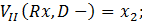  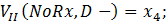 |
| --- | --- |

Overall valuation of decision to treat (Rx) is equal to:


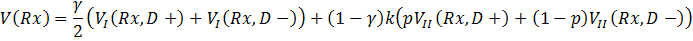


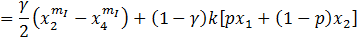


And


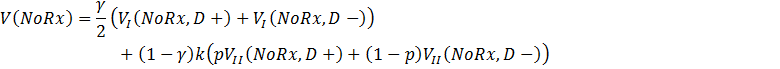


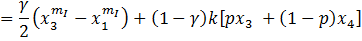


Valuation of the two management strategies will be the same, if


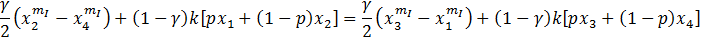


Solving this equation for
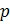
, we have


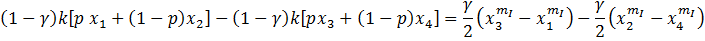


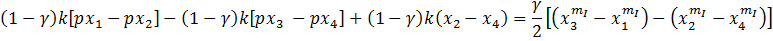


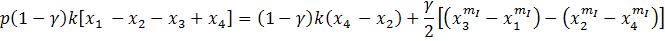


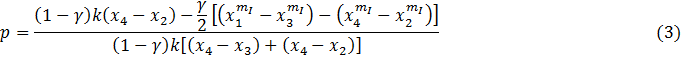


The difference in the outcomes of treating and not treating patient with disease are equal to the net benefit of treatment (B); the difference in outcomes of not treating and treating those patients without disease is defined as net harms (H). Note that benefits and harms can be expressed in the various units (such as survival, mortality, morbidity, costs, etc.) and can be formulated both as utilities and disutilities. As explained above, we further assume that valuation of net benefits and net harms by system I differs from system II. Hence, under system II, we replace net benefit and net harms using EUT definitions:
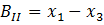
 and net harms
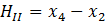
. Under system I, we define
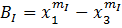
, and
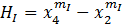
. Solving for *p* (the probability of disease at which we are indifferent between Rx and NoRx), we obtain:


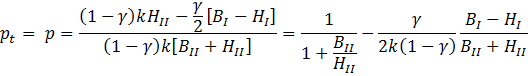


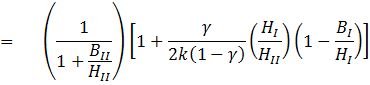


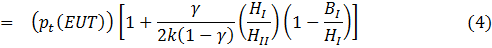


Note that equation 4 may be intuitively better grasped if the net benefits and net harms are expressed using popular clinical summary statistics:


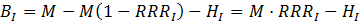


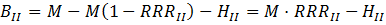


where M=morbidity/mortality without treatment and RRR=relative risk reduction associated with treatment.

Replacing the variables with these new definitions in equation 4, we obtain:


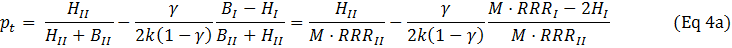


However, as noted in the main text, assessment of benefits and harms by system I are of more qualitative in nature and not as precise as the one used by system II. For example, physicians often assess that “roughly treatment is effective by 90% (meaning that RRR=90%).

**Gamma as a function of the threshold**

It can also be interesting to consider the conditions for involvement of system I as defined by the parameter γ. First, we solve the formula (4) for gamma, i.e., express gamma in terms of the threshold probability:


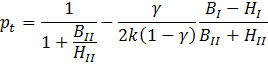


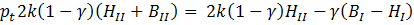


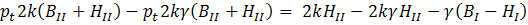


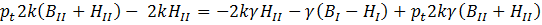


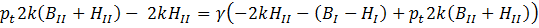


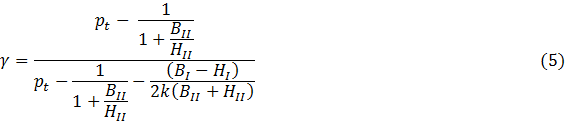


**Thresholds for**
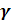


We can also find the threshold for gamma at which both choice options have equal values, i.e., when
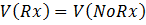
:


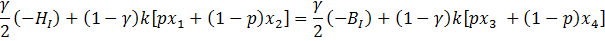


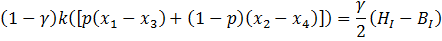


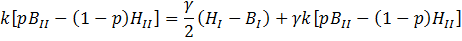


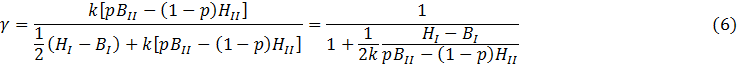


**References:**

1. Djulbegovic B, Hozo I: **Linking Evidence-based Medicine to Clinical Decision Analysis**. *Med Decision Making* 1998, **18**:464 (abstract).

2. Pauker S, Kassirer J: **Therapeutic decision making: a cost benefit analysis**. *N Engl J Med* 1975, **293**:229 -234.

3. Pauker SG, Kassirer J: **The threshold approach to clinical decision making**. *N Engl J Med* 1980, **302**:1109 - 1117.

4. Djulbegovic B, Hozo I, Schwartz A, McMasters K: **Acceptable regret in medical decision making.** *Med Hypotheses* 1999, **53**:253-259.

5. Hozo I, Djulbegovic B: **When is diagnostic testing inappropriate or irrational? Acceptable regret approach**. *Med Decis Making* 2008, **28**(4):540-553.

6. Hozo I, Djulbegovic B: **Will insistence on practicing medicine according to expected utility theory lead to an increase in diagnostic testing?.** . *Medical Decision Making* 2009, **29**:320-322.

7. Hozo I, Djulbegovic B: **Clarification and corrections of acceptable regret model**. *Medical Decision Making* 2009, **29**:323-324.

8. Djulbegovic B, Hozo I, Lyman GH: **Linking evidence-based medicine therapeutic summary measures to clinical decision analysis**. *MedGenMed* 2000, **2**(1):E6.
