# Supplementary material for: Dual processing model of medical decision-making
Source: BMC Med Inform Decis Mak. 2012 Sep 3;12:94. doi: 10.1186/1472-6947-12-94 (PMC3471048; doi:10.1186/1472-6947-12-94)
Supplement: Additional file 2 — Table S1. Evaluation of Behavior of Dual Processing Model for Medical Decision-Making (DSM -M). Threshold probability as a function of individual risk perception. [file 1472-6947-12-94-S2.docx]

**Table S1**

**Evaluation of Behavior of Dual Processing Model for Medical Decision-Making (DSM -M)**

**Threshold probability as a function of individual risk perception**

(For γ=0.5 and various benefit/harms ratio)

| 1. **H_I_/H_II_=10** | |
| --- | --- |
|  | 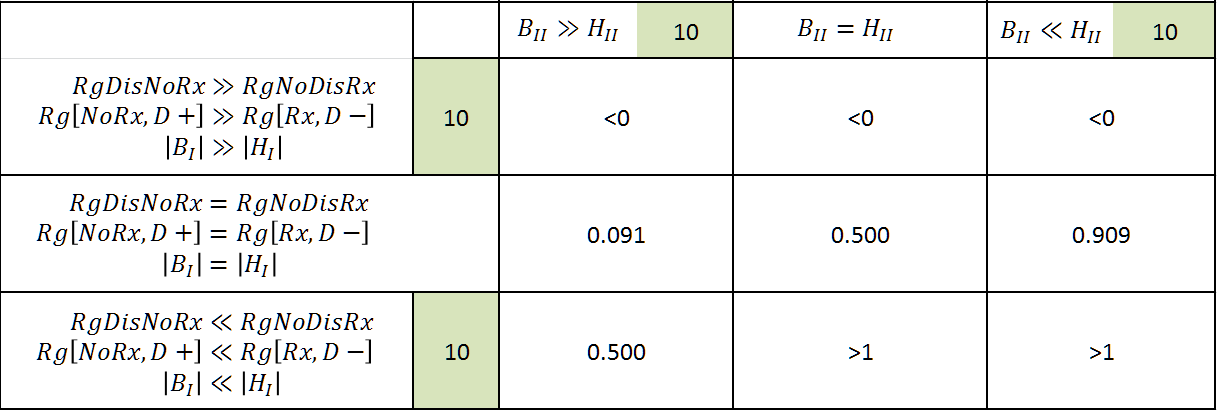 |
|  | |
| 1. **H_I_/H_II_=1** | |
|  | 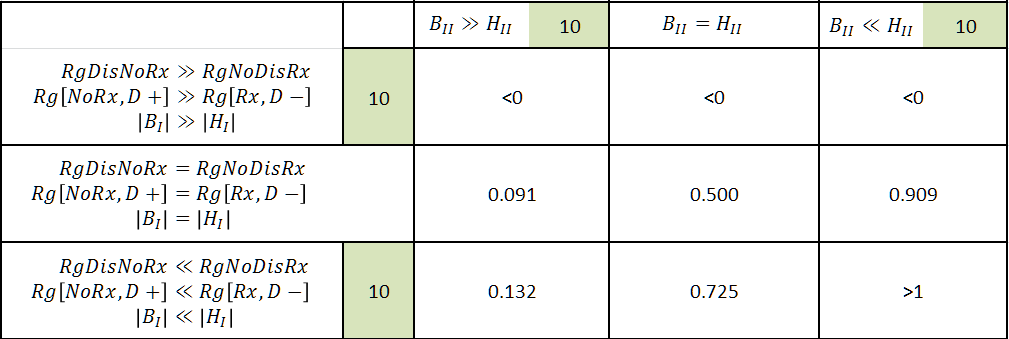 |
|  | |
| 1. **H_I_/H_II_=0.1** | |
|  | 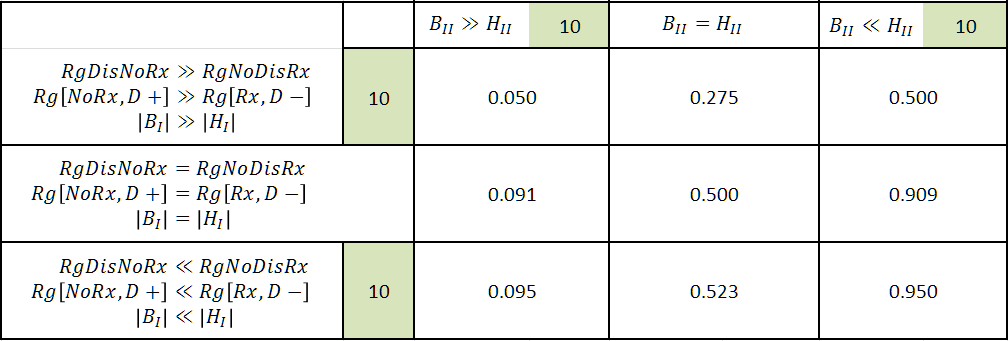 |
